# Towards Trustworthy AI in Healthcare: Epistemic Uncertainty Estimation for Clinical Decision Support

**DOI:** 10.3390/jpm15020058

**Published:** 2025-01-31

**Authors:** Adrian Lindenmeyer, Malte Blattmann, Stefan Franke, Thomas Neumuth, Daniel Schneider

**Affiliations:** 1 Center for Scalable Data Analytics and Artificial Intelligence (ScaDS.AI) Dresden/Leipzig, Leipzig University, Humboldtstraße 25, 04105 Leipzig, Germany; 2 Innovation Center Computer Assisted Surgery (ICCAS), Leipzig University, Semmelweisstrasse 14, 04103 Leipzig, Germany

**Keywords:** uncertainty estimation, epistemic uncertainty, knowledge uncertainty, clinical decision support, electronic health records, trustworthy AI

## Abstract

**Introduction:** Widespread adoption of AI for medical decision-making is still hindered due to ethical and safety-related concerns. For AI-based decision support systems in healthcare settings, it is paramount to be reliable and trustworthy. Common deep learning approaches, however, have the tendency towards overconfidence when faced with unfamiliar or changing conditions. Inappropriate extrapolation beyond well-supported scenarios may have dire consequences highlighting the importance of the reliable estimation of local knowledge uncertainty and its communication to the end user. **Materials and Methods:** While neural network ensembles (ENNs) have been heralded as a potential solution to these issues for many years, deep learning methods, specifically modeling the amount of knowledge, promise more principled and reliable behavior. This study compares their reliability in clinical applications. We centered our analysis on experiments with low-dimensional toy datasets and the exemplary case study of mortality prediction for intensive care unit hospitalizations using Electronic Health Records (EHRs) from the MIMIC3 study. For predictions on the EHR time series, Encoder-Only Transformer models were employed. Knowledge uncertainty estimation is achieved with both ensemble and Spectral Normalized Neural Gaussian Process (SNGP) variants of the common Transformer model. We designed two datasets to test their reliability in detecting token level and more subtle discrepancies both for toy datasets and an EHR dataset. **Results:** While both SNGP and ENN model variants achieve similar prediction performance (AUROC: ≈0.85, AUPRC: ≈0.52 for in-hospital mortality prediction from a selected MIMIC3 benchmark), the former demonstrates improved capabilities to quantify knowledge uncertainty for individual samples/patients. **Discussion/Conclusions:** Methods including a knowledge model, such as SNGP, offer superior uncertainty estimation compared to traditional stochastic deep learning, leading to more trustworthy and safe clinical decision support.

## 1. Introduction

### 1.1. Background

Electronic Health Records (EHRs) encapsulate an immense volume of data, encompassing intricate patient histories, treatment pathways, diagnostic information, and clinical outcomes [1,2]. However, the sheer magnitude and complexity of EHR data is often beyond the capacity of human practitioners to effectively process in its entirety. This limitation necessitates the development of automated methods capable of identify complex patterns, summarizing vast data [3,4,5], and indicating critical points that require human attention [6,7,8].

Machine learning (ML) approaches have shown promising results in the analysis of EHR data in plenty of studies centered around a multitude of predictive clinical applications [9]. In the healthcare domain, where decisions are safety-critical and ethically relevant, it is imperative that such automated methods employed in clinical decision support systems (CDSSs) are not only effective but also trustworthy [6,7,8,10,11]. While many studies focus on certifying predictive performance during model validation on unseen yet familiar in-distribution (ID) data, performance on unfamiliar out-of-distribution (OoD) data remains unchecked and inconsistent [7,12]. Standard ML approaches often naturally extrapolate in an uncontrolled fashion from available data [11], potentially producing confident predictions misleading users into overestimating the evidence supporting a prediction. In the medical context, this is particularly concerning, as it may lead to a false sense of security (or unwarranted concern), potentially influencing a clinicians decision-making inappropriately. The consequence may be false decision-making and decreased quality of medical care [8,10,11,12,13,14]. A striking example is prevailing racial bias in datasets leading to real-world problems for underrepresented populations [14]. CDSSs deployed in real-world applications are very likely to encounter OoD data, but distinguishing between complex ID and OoD data through human observation alone is unfeasible in real-world scenarios [12,14]. The inability to decide which predictions to trust and which not to renders the reliability of every prediction questionable. If there is a possibility that such uncertainty remains undetected, clinicians would need to assume such uncertainty for every sample, leading to a pervasive mistrust in any given prediction. This general distrust may prevent AI from contributing meaningfully to improve patient outcomes, even in cases where they could provide significant clinical value. Hence, it is crucial for CDSSs to convey the extent of the evidence supporting their predictions [6,7,8,10,11,15,16]. This scenario is distinct from cases where there is simply a lot of noise for a given prediction. Stochasticity in the prediction arises from inherent noise within the data or the real world and is not a limitation of the model or evidence itself. In certain patient cases, predictions cannot be more precise. Inconclusive predictions may still be statistically sound and clinically usable. In contrast, predictions not backed by evidence are not practically usable. In such situations, clinicians should disregard the prediction, the uncertainty should be clearly reflected in the prediction or the prediction should not be presented at all to prevent undue influence on clinical decision-making.

### 1.2. Related Work

Early analysis of medical reasoning processes and the wish for computerized support in the medical field has been around since 1959 [17] and uncountable progress has been made in the field ever since [9,18,19]. While many different use cases such as image detection have received a lot of attention even in the medical field, the recent large-scale collection of patient EHR data [20,21] and continued improvements in computational resources has lead to a whole new class of systems entering the medical field [9,22,23]. Tapping into the patients timeline, these models access longitudinal information that is important for clinical decision-making [24]. Models for EHR data need the ability to process sequential data. Its similarity to textual data has lead to the application of models known from the natural language processing (NLP) domain, namely variants of recurrent neural networks (RNNs), Long Short-Term Memory networks (LSTM) and lately the Transformer [19,25,26].

In [27], the authors developed DoctorAI that predicts next-visit diagnosis and medication codes utilizing a classical RNN-based architecture. Building on prior work, they propose RETAIN, an architecture more specifically targeted and inspired by medical decision-making [28]. Two LSTMs are used to direct attention at certain visits and features. Important information pieces are extracted, combined and passed through a classification network. In [29], a set of benchmarks based on the MIMIC3 dataset [30] is introduced and a baseline is established with different LSTM-based architectures. To the authors’ knowledge, these are the only rigorously defined EHR benchmarks including preprocessing that are usable for the objective comparison of models providing predictions for a selected number or medically relevant use cases, and they have been used in multiple studies since [22,31,32]. The authors of [22] apply a Transformer to the [29] benchmarks. Due to the Transformer’s inherent lack of recurrence, temporal information must be specifically given. While in the landmark paper [25], temporal information is given via temporal embedding and many others have relied on this approach, the studies [33,34] developed other explicit methods of including arbitrary temporal information. While in [33] temporal information is included in the attention layer, in [34] time information is directly applied to the tokens. Research in [23] shows how the embedding of medical tokens can be enriched by including information from medical ontologies. Their model is a combination of a graph neural network for the embedding and a BERT-style Transformer for the inclusion of the longitudinal data aspect. Multiple studies by different autors have extensively investigated the perfromance of Transformer-based models [18,35,36] on different large-scale real-world medical datasets, showcasing the performance beyond typical benchmark datasets and potential real-word applications. While in [37] information contained in medical notes is utilized by training two separate models and combining their embedding for various downstream tasks, the authors of [38] focus specifically on semantical differences between in- and outpatients.

However, with the goal of introducing AI into medical practice on a large scale, AI models are not only expected to be performant but also safe and trustworthy under real-world circumstances [7,8,13,15,16,24,31,32]. Among the multitude of additional requirements and concerns for medically applied AI, [16] found a lack of trust from clinicians to be a major limiting factor. While previous methods have demonstrated impressive performance, they lack mechanisms to enhance trust in the predictions they provide. As previously discussed, these methods can confidently make predictions on unseen data, even in cases where such confidence may not be warranted. This overconfidence, coupled with the absence of transparent uncertainty quantification, has understandably reinforced the skepticism of medical professionals regarding the reliability of AI-based predictions in clinical practice. Providing a measure of uncertainty for a given decision can help alleviate such distrust [7,8,31,32]. While reliable uncertainty estimation has been a research focus for AI for a while, it has made its way into the medical space only recently.

In [32], uncertainty is learned through optimization/regularization. The study presented in [31] investigates the capabilities of RNN and Gated Recurrent Unit (GRU)-based architectures to estimate different types of uncertainty. To produce knowledge uncertainties, they use the dropout method and deep ensembles. Using uncertainty information, they are able to significantly boost results when discarding uncertain examples and show correlations between certain data manipulations and resulting uncertainty. As argued in [7], capturing uncertainty is important for the identification and communication of cases where the model’s decision is likely to be questionable and more data should be collected. They tested ensembles of LSTMs as well as different configurations of Bayesian LSTMs. Work in [39] explores the role of uncertainty in enhancing collaborative decision-making in mental health care based on the MIMIC3 dataset. It emphasizes that uncertainty is inherent in clinical environments, particularly in mental health, and highlights significant improvements in performance and safety by referring uncertain predictions to clinicians. The study done in [40] shows how uncertainty impacts clinical decision-making processes by employing a bootstrapped counterfactual inference framework. This methodology allows for the quantification of uncertainty in treatment effects and outcomes, thereby enhancing the robustness of clinical decisions made in the face of incomplete information and variability in patient responses.

Although some studies have underscored the importance of incorporating sample-wise uncertainty, its practical implementation remains uncommon. Broader adoption could significantly enhance ethical standards, address legal considerations, and foster greater trust in decision-support systems.

Detection of OoD samples is intricately linked to the estimation of knowledge uncertainty. Knowledge uncertainty represents the ambiguity in the model function learned from data. Unlike stochastic uncertainty, which stems from inherent data variability and is typically addressed by modern ML approaches, knowledge uncertainty is unrecognized by point estimators or typical single model approaches [11]. In the quest to quantify knowledge uncertainty, common methodologies involve sampling from a functional posterior distribution consistent with the training data [13]. Among these methods, model ensembles and implicit ensembling methods such as dropout or stochastic model parameters stand out as particularly prominent and are as of today widely used for uncertainty quantification in diverse fields [15]. In this work, we critically evaluate the effectiveness of these models in estimating predictive uncertainty within a practical, application-driven CDSS scenario and compare it with a state-of-the-art Neural Gaussian Process approach [41]. This evaluation is pivotal in understanding how these models perform in real-world healthcare settings, where the distinction between ID and OoD data is vital for making reliable clinical decisions. To our knowledge, we are among the first to employ and compare these methods for applications in the medical domain and discuss their behaviour on sound medical decision-making either with or based on AI.

## 2. Materials and Methods

### 2.1. Estimation of Predictive Uncertainty

In the presented context, predictive uncertainty can be divided into stochastic uncertainty (SU) indicating stochastic noise, and knowledge uncertainty (KU) which stems from a lack of knowledge [11]. Given a specific feature set, SU defines the upper limit of certainty for a prediction as the data does not support lower ambiguity. This reflects the stochastic nature of the system—such as overlapping classes, measurement noise, or inherent randomness—making SU proportional to these effects (i.e., high SU for a prediction indicates significant noise). KU is the ambiguity of the correct model function. Assuming certain smoothness constraints, the set of possible functions within tightly sampled regions is small, resulting in little ambiguity of the model function. However, in regions sparsely sampled or far away from known data points, ambiguity about the model function grows rapidly. KU can thus be regarded as a measure of the extent to which the predictions are supported by the evidence [7,42,43] (i.e., high KU indicates little/no data supporting the prediction).

For a classification task, neural networks are SU-aware by design as it is usually gained as a byproduct of the training procedure (likelihood optimization) [7,31], while KU is less easily accessible [7]. Hence, attention is usually directed towards enabling a model to quantify KU. A recent paper [13] reviewed a large number of current methods. In the scope of this paper, we focus on two, a classical, yet still widely used [15], model ensemble approach and the recently developed method of Spectral Normalized Neural Gaussian Processes [41].

#### 2.1.1. Ensemble Neural Networks (ENNs)

**Figure A1:**
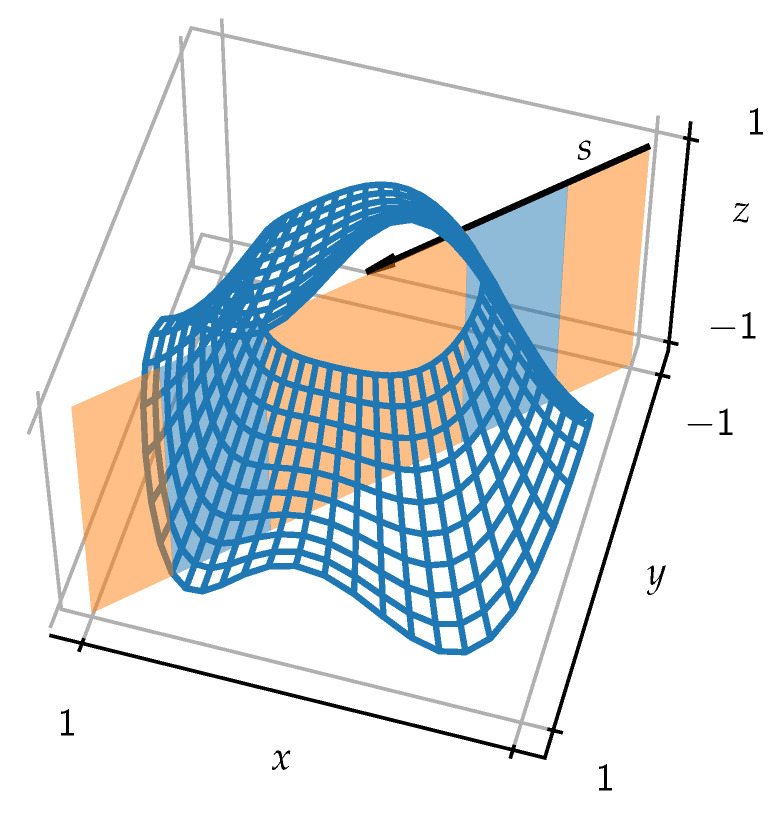

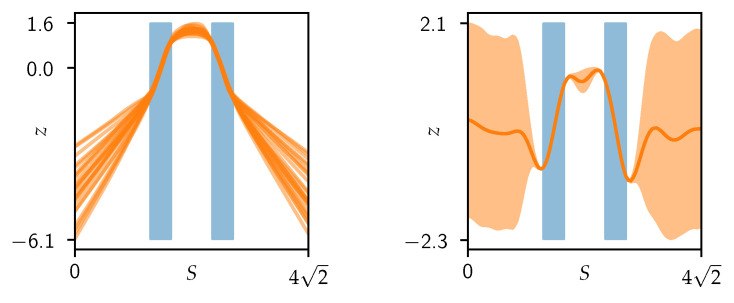
Visual representation of ENN and SNGP general behavior. **Top**: Exemplary problem. **Bottom**: Behavior along the cut plane *s*. The ENN method shows typical diverging behavior resulting in a measurable signal for knowledge uncertainty. The SNGP method shows approximated Gaussian Process behavior with the variance (represented by the orange 95% confidence interval) directly usable as signal for knowledge uncertainty.

An ENN consists of multiple individual models. Due to random initialization, stochastic optimization and high-dimensional loss surfaces, models learn different solutions. The resulting difference in the built functions could theoretically approximate a posterior distribution from which KU can be measured. In areas of high sample density, models will tightly agree on the solution and thus collectively signal low KU. Consequently, in areas of low sample density or for unfamiliar samples, divergent model solutions should signal high KU [13]. Highly over-parameterized models such as neural networks should be especially suitable due to their universal function approximator capability that enables highly dissonant behaviors. A simple illustration is shown in the Appendix A.1 in Figure A1.

We utilize an approach similar to that of [44], making use of heterogeneous models in terms of overall structure, combining smaller and larger models varying the number of layers and layer sizes. Our experiments, classification of low dimensional toy data and mortality prediction, are structured as binary classification tasks. We utilize the functional variance observed in the output space across ensemble members, denoted as σ(L) where L represents the output of the members, as a measure of KU.

#### 2.1.2. Spectral Normalized Neural Gaussian Process (SNGP)

The SNGP was recently introduced in [41] and can be summarized as a Laplace approximated neural Gaussian process with a radial basis function kernel. Spectral normalization of hidden layers preserves distances between data points across the model while a distance-aware Gaussian Process enabled by Random Fourier Features and Laplace approximation calculates a variance measure σ that is proportional to the distance to previously seen points. The output approximates a regular Gaussian process $f\left(x_{q}\right)∼GP\left(0,k\left(x_{q},x_{t}\right)\right)$ resulting in a distribution over predictions $p\left(f\left(x_{q}\right)|X_{t},y_{t}\right)∼N\left(\mu \left(x_{q}\right),\sigma \left(x_{q}\right)\right)$, where *q* indicates the current query and *k* represents the training samples. As such, we use $\sigma (x_{q})$ as a measure for KU. Details of the SNGP go beyond the scope of this work but can be found in the source publication.

### 2.2. Toy Datasets

We feature two different toy datasets: the popular two-dimensional two moons dataset highlighting general behavior and ensuring comparability, and a custom two-dimensional stripes dataset featuring vertical stripes of different data densities, class distributions and gaps of varying sizes between the stripes. Due to the stripes’ vertical orientation, the $x_{2}$ direction is theoretically inert on a macroscopic level. This will allow us to examine the extrapolation behavior in the inert direction and challenges the behavior of classical neural networks due to random edge effects dominating the prediction.

**Figure 1:**
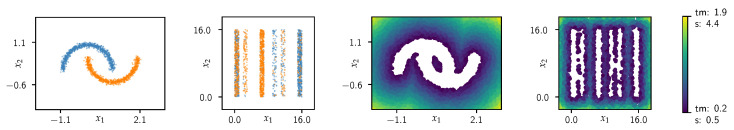
Left: The common two moons dataset and the stripes dataset. Right: Synthetic OoD datasets for both the two moons and the stripes dataset. The colorbar indicates the distance to the nearest known data point from the source dataset.

We also generate a synthetic OoD dataset by producing points that are at least a dataset-dependent length scale away from their nearest data point. For the two moons dataset, we select half the minimum width of the moons (≈0.2), and for the stripes dataset, half the minimum width of the stripes (≈0.5). We utilize these OoD datasets to determine how well the uncertainty measures produced by the different methods are able to differentiate between ID and OoD data, and where OoD points are located that are not properly distinguished from ID points. All datasets are shown in Figure 1.

### 2.3. Medical Dataset

MIMIC3 [30,45,46] is a large publicly available dataset of roughly 40 k patients who where admitted to the ICU at Beth Israel Deaconess Medical Center in Boston, Massachusetts. It includes a wide range of clinically relevant information such as vital parameters, laboratory results, clinical procedures, medications, and outcome measures such as mortality. Recently, a benchmark based on MIMIC3 was introduced in [29]. In their work, the authors delineate preprocessing methodologies, outline cohort selection strategies, identify multiple clinical predictive tasks, and set performance baselines for prevalent deep learning models. Depending on the selected predictive task, the cohort selection is slightly different to accommodate task-specific exclusion criteria and the data is preprocessed according to the needed structure. For a more detailed description of the data and preprocessing steps, see [29].

This work specifically uses the in-hospital mortality prediction task. At its core, the dataset is comprised of 21,139 patients (further selected based on age, completeness of records, minimum length of stay, etc.) and a subset of 17 continuous and discrete features. Episodes begin at the time the patient is admitted to the ICU. Prediction of patient death occuring in-hospital is made 48 hours later. Signals are discretized to an hourly step size.

### 2.4. Medical Data Model for Transformer Application

To effectively process the time-dependent EHR data, we employ an Encoder-Only Transformer model and propose a data model designed to capture various clinical data modalities and unify them in a single embedding space for processing by the Transformer models.

EHR data can be viewed as a longitudinal stream of heterogeneous tokens. The token nomenclature is borrowed from the NLP domain and indeed there are similarities between language information and EHR records that have been exploited by numerous works [9,18,22,34,38]. There are, however, fundamental differences on the token level that need to be addressed [18,33]. Medical tokens are not restricted to words but encompass a multitude of heterogeneous concepts such as diagnostic values, laboratory results, vital parameters, medical imaging data, entire medical notes, medical procedures and medications, as well as data from the omics spectrum. During the presented study, we limit ourselves to singular values, but the concept could be extended to more complex inputs through summarization by upstream models or processing. Addtionally, medical records include a temporal component, in contrast to written language, which relies on information of order only.

For the task of outcome prognosis on EHR time series, we define two types of medical tokens: Boolean (${\mathrm{token}}^{\left(b\right)}$) and value tokens (${\mathrm{token}}^{\left(v\right)}$). Boolean tokens represent singular concepts (i.e., a patient’s verbal response is categorized as confused on the Glasgow Coma Scale (GCS)). Value tokens represent a concept with an attached continuous value (i.e., a measured heart rate at a value of 88 bpm). The token types are shown in Equations (1) and (2). Both incorporate a concept *c* and a timestamp *t*. Value tokens further include a value *v*.

(1) $$\begin{array}{cc} {\mathrm{token}}_{i}^{\left(b\right)} & ={\left(t,c\right)}_{i} \end{array}$$

(2) $$\begin{array}{cc} {\mathrm{token}}_{i}^{\left(v\right)} & ={\left(t,c,v\right)}_{i} \end{array}$$

We employ a standard 1-hot encoding technique for the concept part of both token types. To include the value component, we use a similar “1-value” encoding which is the same as 1-hot but multiplied by the value. To incorporate time, we translate the time stamps to a scalar and utilize the result as input to the circular time embedding also used by the authors of [25].

### 2.5. Medical OoD Dataset

The medical dataset does not allow for a straightforward distance measure, unlike the simpler, toy datasets (see Section 2.2). Determining how far apart patients are from each other is challenging and may not be strictly feasible. This difficulty arises from several factors, including the mix of categorical and continuous features, variations between samples in terms of time span, measurement frequency, and the attributes measured, as well as differences in the relative importance of specific features. For example, a single anomalous heart rate reading does not necessarily equate in significance to an unexpected medication event.

To generate OoD patients, we employ two strategies designed to test distinct scenarios:

**Random Token Replacement (RTR):** Instead of the correct token, we feed random input drawn from N(0,1) into the models. These randomized tokens are highly recognizable at the individual token level.
**Patient Token Swapping (PTS):** We replace tokens with randomly selected tokens form other patients. While these substituted tokens are realistic at the individual token level, the resulting combination of tokens may be unfamiliar only in the context of the remaining patient data.

In both approaches, we require a proxy that correlates with the conceptual distance from known patients. We consider an unmodified patient as a known data point, while a patient in which all tokens are either replaced with random tokens or completely swapped with tokens from other patients represents the maximum possible distance. Consequently, we define a distance measure ranging from 0 to 1, representing the ratio of changed tokens to the total number of tokens in a patient.

(3) $$\begin{array}{c} d_{proxy}=\frac{n_{changed}}{n_{total}} \end{array}$$

where $n_{changed}$ represents the number of tokens changed either by random token replacement or by patient token swapping and $n_{total}$ represents the number of tokens in a patient.

## 3. Results

We conducted a series of experiments in a bottom-up fashion, beginning with small-scale experiments and scaling up to a more complex use case. This approach allowed us to trace the behaviors (predictive and uncertainty estimation) observed in the small-scale experiments. The large-scale experiment, based on the previously introduced MIMIC3 dataset, enables us to demonstrate the implications for informed medical decision-making using uncertainty-enabled AI models.

### 3.1. Toy Data Experiments

A simple ReLU-activated, fully connected feed forward network serves as the backbone of each model. ENN models consist of 27 different architectures. Network depths range between one and three layers and widths of 100–150 neurons. All model outputs are pooled (output space mean and variance; see Section 2.1.1) for a single prediction and uncertainty measure. SNGP results are pooled (output space mean and averaged variance output; see Section 2.1.2) over nine models of comparable depths and widths. All models are trained using Adam and early stopping. Uncertainty measures are transformed using a log(10σ) scale in order to enhance minute details in areas of generally lower uncertainty such as the “interior” of the datasets.

**Figure 2:**
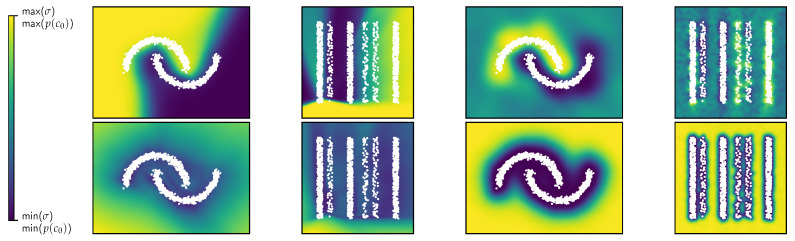
Results from the toy data experiments. **Top**: Class predictions. **Bottom**: KU measures. **Left**: ENN. **Right**: SNGP.

Detailed AUROC and AUPRC performance measures are shown in Appendix A.2 and indicate that all models are generally performing well (AUROC: >0.90, AUPRC: >0.85), as is to be expected for such simple datasets. The similarity across models of both AUROC and AUPRC measures shows that all models solve the underlying problem to equal degrees. The different behaviors in terms of prediction and uncertainty estimation can be seen in Figure 2.

The two methods exhibit distinct differences in prediction and uncertainty estimation, as observed in both the two moons and stripes examples. The ENN-based approach demonstrates a typical decision boundary separating the two moons and, upon closer inspection, a separation between the stripes. However, this separation becomes chaotic at the top and bottom of the stripes dataset, leading to overconfident class predictions if not properly managed by uncertainty estimation.

The SNGP presents a behavior akin to the Gaussian process it is approximating, forming “class islands” surrounded by mean predictions in regions without data. The uncertainty estimation shows a controlled and principled behavior where the distance from but not the topology of the actual data is relevant for the amount of uncertainty in both the experiments, providing a more stable assessment of uncertainty.

In contrast, the ENN’s uncertainty estimation appears to be less controlled, often underestimating uncertainty. This is especially evident in the region between the two moons, where there is an area of low uncertainty. For the stripes experiment, the ENN method displays further issues, with significantly higher uncertainty at the bottom than at the top, which may be influenced by edge effects in the dataset. However, this discrepancy does not align with an intuitive understanding of certainty (based on the distance to the nearest known point), as the stripes dataset is theoretically constant in the $x_{2}$ direction. Thus, no difference in uncertainty between the top and bottom should be expected. This issue contributes to unchecked overconfidence, particularly at the top.

**Figure 3:**
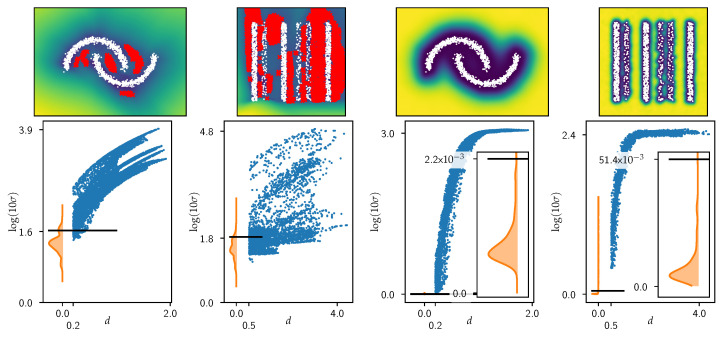
OoD detection experiments on the two moons and stripes datasets. **Top**: KU heat maps with red markers indicating samples from the OoD datasets that are not recognized as OoD by the threshold. **Bottom**: EU values of the test dataset are shown as a kernel density estimate ($p_{test}(\log\left(10\sigma \right)$) in orange together with the threshold (black line) based on the 90th quantile. KU values of the OoD samples are shown as blue dots. **Left**: ENN. **Right**: SNGP.

We evaluate the estimated uncertainty based on the nearest neighbor (NN) distance from the respective ID datasets (see Figure 3). To distinguish between ID and OoD samples, we chose a threshold derived from the KU measures recorded on the test splits of the training data for the two datasets. Specifically, we set the threshold at the 90th percentile of these KU values (horizontal line in Figure 3 plots), meaning that 10% of the test samples were classified as OoD, while the remaining 90% were considered ID. While this has no deeper reason, a higher threshold would result in even more undetected OoD samples and a lower threshold would result in the classification of more test samples as OoD, which is undesirable.

The results reveal that the ENN-based model struggles to effectively differentiate between ID and OoD samples, even in these simple scenarios. For the two moons example, there are clear instances of OoD detection failures, particularly in the region between the two moons. The stripes dataset presents an even more pronounced issue, with the model failing to reliably identify OoD samples inside and outside the stripes. Notably, in the regions between the stripes, almost none of the OoD samples are detected using the established threshold.

The notion that some OoD examples may be indistinguishable from test samples is challenged by the performance of the SNGP model, which successfully differentiates between OoD samples and test samples without any detection failures.

The code used for this analysis is provided in the Supplmentary Material.

### 3.2. MIMIC Mortality Prediction

For the SNGP and ENN methods, we train a collection of models and aggregate their results. In the ENN-based approach, we average the outputs to generate the overall prediction and derive an uncertainty measure based on the deviation among the model predictions (σ(L)) (see Section 2.1.1). For the SNGP-based approach, we average both their prediction and uncertainty outputs (σ) (see Section 2.1.2).

All methods utilize a common backbone structure of an Encoder-only Transformer. The network depths range from two to four layers, with widths between 64 and 512 neurons. However, due to computational limitations, we do not include models that are both wide and deep (e.g., four layers with 512 neurons).

For the SNGP models, all feed-forward layers are modified to be bi-Lipschitz (meaning distance preserving up to a constant factor), as outlined in the source publication [41] to preserve distances between data points. As there is currently no established method for bi-Lipschitz Attention mechanisms, we employ the standard Attention mechanism as a fallback for all models.

We ensemble eight different models for the ENN approach, varying in model sizes and architectures. For the SNGP-based approach, we ensemble four models, also spanning various sizes and architectures.

We study model performance on the in-hospital mortality task from the [29] benchmarks (see Section 2.3) which uses 48 h of patient intensive care signals and predicts if death occurs within the current hospital stay. For further details, we refer the reader to the source publication. Our two models are compared against performance metrics (AUROC and AUPRC) of two models published in the benchmark [29], including the Standard-LSTM (S-LSTM), trained in a similar fashion to our two models on only the in-hospital mortality use case. Secondly, the Multitask Channel-wise LSTM Model (MTCW-LSTM) was selected as it represents the best model published with the benchmark while not being entirely comparable due to its multi-task training.

**Figure 4:**
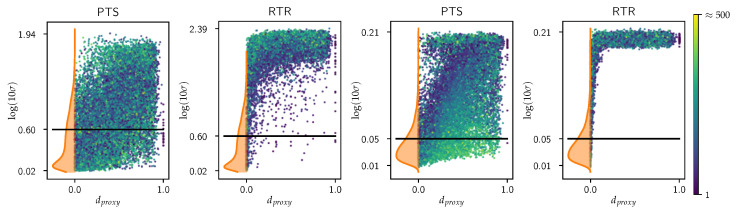
OoD detection experiments PTS and RTR on the MIMIC3 benchmark dataset. **Left**: ENN. **Right**: SNGP. Uncertainty measures of the test dataset are shown as the kernel density estimation ($p_{test}(\log\left(10\sigma +1\right)$) in orange together with the threshold (black line) based on the 70th quantile. KU values of the OoD samples are shown as points. Points are colored according to the number of tokens for each sample.

To assess the robustness of the ENN- and SNGP-based approaches, we apply the same OoD detection experiment that was previously conducted on the toy datasets. By setting a threshold derived from the uncertainty measures of the ID test samples, we analyze the proportion of OoD examples that are not captured by this threshold. Finally, we provide an analysis of which OoD samples are detected by the threshold and explore the underlying reasons for their successful identification. Results are shown in Figure 4.

The performances of the ENN and SNGP models together with reference models from [29] in terms of AUROC and AUPRC metrics are shown in Table 1. All models reached an AUROC of ≈0.85 and an AUCPR of ≈0.52. While both the ENN and the SNGP slightly outperform the S-LSTM, their performance falls marginally short of the MTCW-LSTM. The MTCW-LSTM, however, was trained on multiple tasks and is only shown as it represents the best model published in [29]. As the observed performance differences fall well within the calculated confidence intervals, we conclude that the models exhibit comparable performance and SNGP does not significantly decrease performance compared to the ENN model.

For the large-scale outlier detection experiment, we applied a threshold at the 70th percentile of the uncertainty values within the test set, taking into account a lower tolerance for uncertainty in safety-critical domains. Using this threshold for OoD detection, Figure 4 and Table 2 show that the SNGP significantly outperformed the ENN, as indicated by the much lower percentage of altered (RTR and PTS) patients unrecognized by the applied threshold. Both ENN- and SNGP-driven models demonstrated a high degree of success in detecting RTR outliers, with the ENN missing approximately 1.62% and the SNGP missing <0.7%. Despite these strong results, the ENN method exhibited instances of failure to detect distant outliers, while the SNGP showed flawless detection, particularly for larger $d_{proxy}$ values. For $d_{proxy}$ values greater than 0.5, the ENN method continued to fail to detect some RTR patients, even though these patients were at least 50% composed of random input tokens.

The most significant and practically relevant differences between the ENN and the SNGP were observed in the detection of PTS outliers. Both methods missed a substantial proportion of PTS outliers, but the SNGP, which missed 22.3% of PTS patients, demonstrated an increase in detection performance over the ENN, which missed approximately 33.7%.

## 4. Discussion

While the SNGP and the ENN had similar predictive performance, the SNGP demonstrated a superior capacity for OoD detection in both toy experiments and the real-world predictive case study of ICU mortaility prediction. OoD detection with the ENN was less correlated to the degree of modification from the original EHR sequences. Translating this behavior to the medical domain implies that such a predictor could overlook critical discrepancies within a patient’s history—an outcome that is clearly undesirable. In contrast, the SNGP was considerably more reliable, detecting OoD samples with randomized feature values in the RTR experiment, even when alterations were relatively minor.

In the PTS experiments, discrepancies in altered patients are much more subtle, manifesting primarily across the patient’s overall history rather than at the individual token level. This presents a much more challenging, but also more clinically relevant, scenario. In this context, the differences between the ENN- and SNGP-based approaches became evident primarily by contrast, with the SNGP detecting ≈11% more PTS patients than the ENN. Translating this to the medical domain results in an SNGP-driven uncertainty detector that is not only more sensitive at the token level but also at the inter-feature and sequence level across the entire patient’s history.

While an in-depth investigation is outside the scope of this work, several reasons why the SNGP, despite its strong performance in the toy data experiments, missed a significant proportion of PTS samples and performed worse for longer sequences in the large-scale experiment could be because of several factors:

1. Data-induced effects:
  (a) The MIMIC dataset is highly repetitive, composed largely of frequently monitored variables such as heart rate, blood pressure, and oxygen saturation. If such tokens are swapped, the overall consistency of the patient representation may not deviate significantly from known samples.
  (b) The $d_{proxy}$ distance measure is only weakly correlated to epistemic uncertainty. Thus, the true distance is not precisely captured, leading to a potentially fuzzy relationship.
  (c) The discrepancy between long and short timelines may stem from the fact that swapping tokens also swaps their associated timestamps. In short timelines, a swapped token from a long timeline may be more noticeable due to the time signal, whereas in long timelines, this effect is less pronounced, leading to decreased sensitivity.
2. Model-induced effects:
  (a) The SNGP-driven models for the toy data experiments consisted entirely of bi-Lipschitz continuous layers, meaning a mathematically guaranteed preservation of distances across the model. As previously mentioned, there is currently no widely accepted bi-Lipschitz continuous Attention block driving the Transformer models used within the experiments involving EHRs. This could also lead to the model suppressing subtle distinctions by minimizing the impact on the larger patient mortality prediction and as such on the uncertainty prediction.
  (b) While Transformer models are capable of processing inputs of varying lengths, in most architectures, the final prediction is derived from some form of summary representation (e.g., a specialized token, an average over all tokens, etc.). In our architecture, we employ the averaged-token approach. However, this operation is inherently not distance-preserving, which means that crucial information regarding the relative distances between patients may be lost during this summation process.

While this study provides valuable insights and demonstrates clear advantages of the SNGP over ENNs that rely on random training dynamics, several limitations must be acknowledged. Our analysis was restricted to relatively simple ensemble methods, despite the existence of more advanced approaches that could further enhance performance. Incorporating these modern ensemble techniques may yield additional improvements in uncertainty estimation.

Additionally, while the generated OoD datasets serve as a useful tool to assess the model’s ability to delineate unfamiliar data, their practical relevance to real-world patient populations remains unknown. The synthetic nature of these datasets may introduce unrealistic patient profiles, potentially featuring mutually exclusive attributes that are unlikely to co-occur in clinical settings. In practice, such inconsistencies are typically attributable to erroneous data entries rather than inherent pathological characteristics.

Future research could address model-induced artifacts by refining the underlying mechanisms. Notably, the current self-attention mechanism employed in our approach is not Lipschitz continuous, as demonstrated in [47]. However, recent advancements have introduced alternative formulations that uphold Lipschitz continuity [47,48,49,50], offering promising avenues for enhancing SNGP Transformers. Implementing these methods may contribute to more robust and reliable uncertainty quantification in medical decision support systems.

## 5. Conclusions

Recognizing the need for trustworthy and reliable AI in safety-critical clinical decision-making, we investigated the KU estimation performance of an ENN, and compared its accuracy to the KU quantification of a novel method, a SNGP. Several differences between ENN- and SNGP-driven models were highlighted in this work, particularly in terms of prediction behavior, uncertainty estimation, and the resulting capability to detect OoD samples. A bottom-up approach was employed, first showcasing distinct behaviors through experiments on toy datasets and then tracing findings to a large relevant prediction task on EHR data.

Our results indicate that the ID predictive performance remains unaffected by the use of a SNGP, demonstrating comparability to an ENN and models reported in other comparative studies. However, the estimation of KU varies significantly between the two models, with the ENN exhibiting a pronounced tendency to underestimate KU—both in toy datasets and in the case study experiments. The SNGP consistently outperformed the ENN, particularly for highly modified patients in both the RTR and PTS experiments. In certain instances, the ENN failed to detect any KU for patients composed almost entirely of random inputs or tokens from other patients, whereas the SNGP demonstrated sensitivity in such scenarios.

The deployment of AI models in the medical domain presents significant implications for sound medical decision-making, with trustworthiness being a key challenge that continues to hinder widespread adoption. A critical concern is the inability of commonly used AI models to reliably detect their own ignorance, which severely undermines trust in all predictions, thereby diminishing their practical utility.

A robust mechanism for distinguishing between predictions that can be trusted and those that cannot would significantly enhance the overall trustworthiness, acceptance, and utility of AI models in medical practice. We showed on the clinically relevant use case of mortality prediction on EHR data that deep learning methods incorporating a specific mechanism or model to represent the amount of knowledge such as an SNGP are a promising step forward to more reliable and trustworthy AI application in clinical decision-making.

## Figures and Tables

**Table 1:** Predictive performance on MIMIC Mortality Prediction Benchmark [29].

Model	AUROC (min/max) ↑	AUPRC (min/max) ↑
ENN	0.858 (+0.00868/−0.0125)	0.532 (+0.031/−0.0197)
SNGP	0.855 (+0.00839/−0.0142)	0.521 (+0.0268/−0.0184)
S-LSTM [29]	0.855 (+0.018/−0.02)	0.485 (+0.052/−0.054)
MTCW-LSTM [29]	0.87 (+0.017/−0.018)	0.533 (+0.051/−0.053)

**Table 2:** OoD detection performance in terms of % undetected OoD samples in PTS and RTR experiments.

Model	Undetected PTS Samples % (min/max) ↓	Undetected RTR Samples % (min/max) ↓
ENN	33.7 (+1.67/−4.12)	1.62 (+0.214/−0.429)
SNGP	22.3 (+0.697/−0.791)	0.615 (+0.11/−0.0725)

## Data Availability

The MIMIC3 database is available at https://physionet.org/content/mimiciii/1.4/ (accessed on 2 December 2024). Preprocessing for the MIMIC3 database can be found in [29]. Code files (including model definitions, training procedure and evaluation metrics) will be uploaded to a git repository to which access will be granted upon request.

## Supplementary Materials

Code for the Toy Data Experiments can be downloaded at: https://www.mdpi.com/article/10.3390/jpm15020058/s1.

## Abbreviations

The following abbreviations are used in this manuscript:

AI	Artificial Intelligence
ENN	Ensemble Neural Network *or* Neural Network Ensemble
EHR(s)	Electronic Health Record(s)
SNGP	Spectral Normalized Neural Gaussian Process
AUROC	Area Under the Receiver Operating Characteristic Curve
AUPRC	Area Under the Precision–Recall Curve
ML	Machine Learning
CDSS	Clinical Decision Support System
ID	In-distribution

OoD	Out-of-distribution
NLP	Natural Language Processing
RNN	Recurrent Neural Networks
LSTM	Long Short-Term Memory
GRU	Gated Recurrent Unit
SU	Stochastic Uncertainty
KU	Knowledge Uncertainty
GCS	Glasgow Coma Scale
bpm	Beats per Minute
S-LSTM	Standard LSTM
MTCW	Multitask Channel-wise LSTM
RTR	Random Token Replacement
PTS	Patient Token Swapping

## Appendix A

### Appendix A.2. Small Scale Performance Metrics

**Table A1:** AUROC and AUPRC performance measures on the two moons and stripes dataset.

	Two Moons		Stripes	
Model	AUROC ↑	AUPRC ↑	AUROC ↑	AUPRC ↑
ENN	0.93	0.93	0.85	0.87
SNGP	0.92	0.92	0.86	0.88
